# 

**Published:** 1878-04

**Authors:** 


### CALIFORNIA STATE DENTAL ASSOCIATION.

The ninth annual session of the California State Dental Association will be held in San Francisco, commencing Tuesday, June 4th, 1878, at ten o'clock, a. m., and continue four days.

### STANDING COMMITTEES.

By-Laws, Art. 2, Sec. 3. * * "Each member may make an individual report, and in case of inability to be present when the report is due, can forward it to the Secretary, from whom it can be obtained by the Chairman of each committee respectively."

Dental Pathology and Surgery.—Drs. Dennis, Dunbar and Davenport.

Dental Therapeutics.—Drs. Asay, Menefee and Whipple.

Dental Chemistry.—Drs. Robinson, Harding and Spencer.

Dental Histology and Microscopy.—Drs. Knowles, (S. E.), Davis, (C. E.) and Ogden.

Operative Dentistry.—Drs. Goddard, Griswold and Gaston.

Mechanical Dentistry.—Drs. Lane, Robinson and Gordon.

Dental Literature and Education.—Drs. Dunbar, Ralls and Goe.

Dental Ethics.—Drs. Dutch, Crawford, Jenkins, Light and Cornwall.

Publication.—Drs. Kingsbury Plomteaux, Hoadley, (ex-officio), Bunnell and Morton.

SPECIAL COMMITTEE.

Dental College.—Drs. Dennis, Knowles, (C. C.), Thomp-Menefee, Dutch, Knox, Younger, Knowles, (S. E.), Bunnell and Plomteaux.

THESIS.

The use of the Microscope. J. L. Asay, M. D.

What is to be the future status of dentistry? Clark A. Goddard, D. D. S.

Professional Etiquette. Wm. Dutch, D. D. S.

The Diseases of the Gums and Process, and their Treatment. S. E. Goe, D. D. S.

The best Methods and Materials for Treating and Filling Children's Teeth. C. S. Lane.

The best Methods of Separating Teeth. S. E. Knowles, M. D., D. D. S.

Prevention and Treatment of Decay on the Proximate Surface of Teeth. Wm. F. Thompson, M. D., D. D. S.

All members are respectfull invited to present voluntary essays on any subject pertaining to our profession which they may select.

Wm. B. Kingsbury, President.
C. S. Lane, Vice-President.
H. J. Plomteaux, Secretary.
S. E. Goe, D. D. S., Cor. Secretary.
J. J. Birge, Treasurer.
H. E. Knox, D. D. S., Librarian.

### ILLINOIS STATE DENTAL SOCIETY.

The annual meeting of the Illinois State Dental Society will convene at Rockford, Ill., May 14th, at ten o'clock a. m.

Arrangements have been completed which will make this one of the most interesting and instructive meetings of this society.

C. R. E. Koch, President, 67 Washington St., Chicago.
S. M. Sturgiss, Vice-President.
E. B. David, Librarian.
E. D. Swain, Secretary, 67 Washington St., Chicago.
C. A. Kitchen, Treasurer, Rockford.
Geo. A. Cushing, Chairman Executive Committee, 174 State St., Chicago.
M. S. Dean, Chairman Board Censors, 174 State St., Chicago.

### EASTERN INDIANA DENTAL ASSOCIATION.

The semi-annual meeting of the Eastern Indiana Dental Association will be held in Knightstown, Henry County, Indiana, commencing Tuesday, May 14th, 1878, at ten o'clock, a. m., and continuing two days.

The Executive Committee would respectfully request a full attendance of the members.

A cordial invitation is extended to all in the profession to attend these meetings.

SUBJECTS FOR DISCUSSION.

First. Relation of Dentists to their Patients.

Second. Are rapid operations consistent with ease to patients and excellence of work?

Third, Devitalized Teeth, and their management.

Fourth. Use of Anæsthetics in Dental Practice.

Fifth. Microscopy.

Sixth. Sensitive Dentine, and its Treatment.
Seventh. Operative and Mechanical Dentistry.

M. H. Chappell, President.
W. A. Rawles, Vice-President.
W. C. Stanley, Treasurer.
J. K. Jameson, Secretary.
J. W. Jay,
J. R. Clayton,
T. S. Hacker,
Executive Committee.

*THE DENTAL REGISTER,*

A MONTHLY MAGAZINE DEVOTED TO THE INTERESTS OF

THE DENTAL PROFESSION.

*Terms Reduced to $2 50 per Year. Single Copies 25c.*

*EDITED BY PROF. J. TAFT, D. D. S.,*

AND PUBLISHED BY

SPENCER & CROCKER, Ohio Dental Depot.

The volume commences in January and closes in December.

The Register being issued monthly is always fresh, therefore Indispensable to the practitioner who desires to keep posted up with the new inventions and improvements which are daily developed. Neither expense nor effort will be spared to give value and variety to its contents. Articles will be illustrated with well executed engravings whenever they will add to the interest or assist to explanation.

Its extensive circulation and frequency of issue make it one of the BEST DENTAL ADVERTISING MEDIUMS in the United States.

All subscriptions must begin with January or July.

Local or special notices, five lines of less, will be inserted for $3.00 each insertion; over five lines, 50 cts. per line.

All advertising bills payable in advance, or at furthest, after the issue of the first number containing the advertisement. All transient advertisements to be paid for in advance.

☞ CONTRIBUTIONS SOLICITED.

Exclusively Editorial Communications should be addressed to Editor.

The latest number of the Register may be ordered with or without other goods at any time, and all letters should be addressed to

**SPENCER & CROCKER, Ohio Dental Depot, Cincinnati, O.**

